# CRAFTED: An exploratory database of simulated adsorption isotherms of metal-organic frameworks

**DOI:** 10.1038/s41597-023-02116-z

**Published:** 2023-04-20

**Authors:** Felipe Lopes Oliveira, Conor Cleeton, Rodrigo Neumann Barros Ferreira, Binquan Luan, Amir H. Farmahini, Lev Sarkisov, Mathias Steiner

**Affiliations:** 1 IBM Research, Av. República do Chile, 330, CEP 20031-170 Rio de Janeiro, RJ Brazil; 2 Department of Organic Chemistry, Instituto de Química, Universidade Federal do Rio de Janeiro, Rio de Janeiro, RJ Brazil; 3 Department of Chemical Engineering, Engineering A, the University of Manchester, Manchester, M13 9PL United Kingdom; 4 IBM Research, 1101 Kitchawan Road, Yorktown Heights, 10598 NY United States of America

**Keywords:** Atomistic models, Metal-organic frameworks

## Abstract

Grand Canonical Monte Carlo is an important method for performing molecular-level simulations and assisting the study and development of nanoporous materials for gas capture applications. These simulations are based on the use of force fields and partial charges to model the interaction between the adsorbent molecules and the solid framework. The choice of the force field parameters and partial charges can significantly impact the results obtained, however, there are very few databases available to support a comprehensive impact evaluation. Here, we present a database of simulations of CO_2_ and N_2_ adsorption isotherms on 690 metal-organic frameworks taken from the CoRE MOF 2014 database. We performed simulations with two force fields (UFF and DREIDING), six partial charge schemes (no charges, Qeq, EQeq, MPNN, PACMOF, and DDEC), and three temperatures (273, 298, 323 K). The resulting isotherms compose the **C**harge-dependent, **R**eproducible, **A**ccessible, **F**orcefield-dependent, and **T**emperature-dependent **E**xploratory **D**atabase (**CRAFTED**) of adsorption isotherms.

## Background & Summary

Carbon capture, storage, and utilization is considered as one of the key strategies required to reduce anthropogenic carbon dioxide emissions and their impacts on climate change^1^. The most viable option for this approach is to focus on capturing CO_2_ from point sources, such as fossil fuel power plants, fuel processing plants and other industrial plants where carbon capture technology can be applied to streams with industrial scale flow rates^2^. However, despite several decades of intensive research, carbon capture in an economically viable way remains an enormous challenge^3^.

Adsorption processes are considered to be a promising alternative to the conventional absorption processes due the their low regeneration energy, high selectivity, and high capture capacity^4^. Combined, these characteristics may lead to energy-efficient processes for industrial scale capture and utilization of greenhouse gases (GHG). At the heart of a typical adsorption process for gas separation, such as the Pressure Swing Adsorption process, is the active adsorbent material; and the efficiency of the process crucially depends on the properties of this material. Within the different adsorbent materials that are potentially available for this process, crystalline nanoporous materials such as metal-organic frameworks (MOF)^5–8^, covalent organic frameworks (COF)^9–11^, zeolitic imidazolate frameworks (ZIF)^12–14^, and zeolites^15^ feature many of the necessary characteristics for a solid sorbent for efficient gas separation under the conditions of interest.

These families of materials contain hundreds of thousands synthesized structures and countless more hypothetical ones, featuring pores of different size, shape, and chemical characteristics. This creates large exploration space for studies that seek to identify the best candidates for a given gas capture application. This endeavour, however, is not possible via a brute-force experimental campaign. The number of large databases built upon experimental^16–22^ and hypothetical^23,24^ structures, combined with the continuous growth of diversity and scope of new materials due to the advancements in digital reticular chemistry^25,26^, make high-throughput computational screening (HTCS) approaches an imperative strategy for efficient exploration of the vast chemical landscape of crystalline nanoporous adsorbents^27,28^.

Most of the HTCS studies for carbon capture and related problems are based on using Grand Canonical Monte Carlo (GCMC) simulations to generate adsorption data. This data is then used to form some simple material performance metrics or is passed on to the process level modelling to explore the performance of candidate materials under realistic process conditions.

To perform molecular simulations such as GCMC, one needs a set of parameters that describe the interactions among the adsorbate molecules, and between the adsorbate molecules and the atoms of the adsorbent material; this set of parameters is called a force field.

Over the years, many force fields have been developed for various purposes and several options are available to describe adsorption of gases such as carbon dioxide in materials such as MOFs. Invariably, the predicted equilibrium adsorption data and, consequently, the ranking of materials and the recommendations of the screening study will depend on the choice of the force field.

This poses several fundamental questions. How sensitive is the adsorption data to the choice of the force field parameters? How does this sensitivity vary across different categories or classes of materials? And ultimately, is a ranking of porous materials for a particular application a robust result or is it contingent on using a particular force field?

To start to explore these questions one needs a representative mass of adsorption data covering typical choices of the force field parameters, materials, gases and conditions. This defines the remit of the current article where we tasked ourselves with building such a database (or at least, the first block of conditions).

To explain the contents of the database and our approach, let us delve first into components of the classical force fields and the typical options available for the studies of adsorption of gases in MOFs and related materials. In the classical force fields, the non-bonded interactions are modeled as a sum of van der Waals and Coulomb potentials^29^. The van der Waals interactions between the adsorbed molecules and the framework are usually modeled by the Lennard-Jones (LJ) potential, which is an effective potential with two fitted parameters that can capture most of the intermolecular effects relevant to physisorption. The parameters for the atoms can be taken from generic force fields such as the Universal Force Field (UFF)^30^, DREIDING^31^ and TraPPE^32^, with the interactions between different atom species computed using mixing rules such as Lorentz-Berthelot^33^ or Jorgensen^34^.

The Coulombic interactions are modeled by partial atomic charges assigned to the atoms which need to be calculated for each material. There are several charge assignment methods available, and they can be divided into three main groups: *i*. methods derived from quantum chemistry calculations (e.g. RESP^35^, CHELP^36^, REPEAT^37^, and DDEC^38–40^); *ii*. methods based on charge equilibration (e.g. Qeq^41^, PQeq^42^, EQeq^43^, and FC-Qeq^44^); and *iii*. methods based on machine learning algorithms, such as PACMOF^45^ and MPNN^46^, that are trained to reproduce the quantum chemistry-based charges but at a much lower computational cost.

Lately, there have been several studies evaluating the accuracy of different methods for calculating partial atomic charges^47–52^, however, little is known about the combined impact of force field and partial charge selection on material-level analysis and its implication on process-level performance metrics. Despite the fact that the deficiencies of charge equilibration methods have already been recognised in the scientific literature, these methods are still commonly used in HTCS studies either as a pre-screening approach^53,54^ or as the final choice of charge assignment method in MOFs^55–57^ due to their simplicity and computational efficiency. Furthermore, the original Lennard-Jones force field parameters were derived and validated using specific partial charge schemes (Qeq^30^ for UFF and Gasteiger^58^ for DREIDING), thus the combination of these parameters with different charge assignment methodologies, even if more accurate, may not necessarily generate better results. Hence, none of the typical combinations of molecular-level modelling choices used in the simulation community (e.g., DREIDING + DDEC, UFF + DDEC, etc.) can be considered as systematically developed or validated and cannot be expected to produce accurate adsorption predictions. These considerations guide us on the choices of the parameters to consider in the database.

The database contains simulated adsorption isotherms for 690 MOFs selected from the CoRE MOF 2014^16,17^ database. The simulations were performed for the adsorption of CO_2_ and N_2_ with two force fields (UFF and DREIDING), six partial charge schemes (no charge, Qeq, EQeq, PACMOF, MPNN, and DDEC), at three temperatures (273, 298, 323 K). The resulting isotherms compose the **C**harge-dependent, **R**eproducible, **A**ccessible, **F**orcefield-dependent, and **T**emperature-dependent **E**xploratory **D**atabase (**CRAFTED**)^59^ of adsorption isotherms. CRAFTED provides a convenient platform to explore the sensitivity of simulation outcomes to molecular modeling choices at the material (structure-property relationship) and process levels (structure-property-performance relationship).

## Methods

### Structure selection

Starting from the 2932 structures present in the CoRE MOF 2014^16,17^, first a set 726 structures that can be simultaneously modelled by both UFF and DREIDING force fields were selected. From these structures, 36 structures were removed due to the presence of unbound water molecules, counter-ions and/or hydrogen atom with incorrect bond lengths/angles. Therefore, a subset of 690 materials that can be modelled by all force fields and partial charge models was obtained and will be referred to as “CRAFTED structures”.

### Partial charges calculation

The DDEC partial charges^38–40^ were taken without modification from the CoRE MOF 2014^16,17^ database. The EQeq partial charges^43^ were calculated using the the extended charge equilibration method as implemented in the EQeq software v1.1.0 (https://github.com/danieleongari/EQeq). The Qeq partial charges^41^ were calculated using the default implementation available in RASPA^60^ v2.0.45. The PACMOF partial charges^45^ were calculated using the default Python implementation available in the PACMOF package (https://github.com/arung-northwestern/pacmof). The MPNN partial charges^46^ were calculated using the MPNN package (https://github.com/SimonEnsemble/mpn_charges).

### Grand canonical Monte Carlo simulations

Atomistic Grand Canonical Monte Carlo (GCMC) simulations were performed using a force field-based algorithm as implemented in RASPA^60,61^ v2.0.45. Interaction energies between non-bonded atoms were computed through a combination of Lennard-Jones (LJ) and Coulomb potentials

1 $${U}_{ij}({r}_{ij})=4{{\rm{\varepsilon }}}_{ij}\left[{\left(\frac{{{\rm{\sigma }}}_{ij}}{{r}_{ij}}\right)}^{12}-{\left(\frac{{{\rm{\sigma }}}_{ij}}{{r}_{ij}}\right)}^{6}\right]+\frac{1}{4\pi {{\rm{\varepsilon }}}_{0}}\frac{{q}_{i}{q}_{j}}{{r}_{ij}}$$

where *i* and *j* are interacting atom indexes and *r*_*ij*_ is their interatomic distance. *ε*_*ij*_ and *σ*_*ij*_ are the well depth and diameter, respectively. The LJ parameters between atoms of different types were calculated using the Lorentz-Berthelot mixing rules

2 $${\varepsilon }_{ij}=\sqrt{{\varepsilon }_{ii}{\varepsilon }_{jj}},\quad \quad {\sigma }_{ij}=\frac{{\sigma }_{ii}+{\sigma }_{jj}}{2}$$

LJ parameters for framework atoms were taken from Universal Force Field (UFF)^30^ or DREIDING^31^ (see Table 1). The parameters for the adsorbed molecules were taken from the TraPPE^32^ force field (see Table 2). All simulations were performed with 10,000 Monte Carlo cycles. Swap (insertion or deletion with with a probability of 50% for each), translations, rotations, and re-insertions moves were tried with probabilities 0.5, 0.3, 0.1, and 0.1, respectively. To avoid the use of long initialization cycles, each isotherm was calculated in a single simulation, with each pressure point of the simulation starting from the result of the previous one. The uptake values for each pressure were obtained by averaging over the GCMC equilibrium phase, determined using the Marginal Standard Error Rule. For more information, please refer to section *Automatic transient regime detection and truncation*.

**Table 1:** Lennard-Jones parameters for UFF and DREIDING force fields.

Atom type	UFF		DREIDING	
	*σ* (Å)	*ε* (K)	*σ* (Å)	*ε* (K)
H	2.571	22.14	2.84642	7.64936
B	3.638	90.51	3.58141	47.80852
C	3.431	52.8	3.47299	47.85884
N	3.261	34.7	3.26256	38.95136
O	3.118	30.2	3.03315	48.16079
F	2.997	25.14	3.0932	36.48545
Cl	3.516	114.15	3.51932	142.57003
Br	3.732	126.22	3.51905	186.20159
Al	4.008	253.94	3.91105	156.00674
Si	3.826	202.15	3.80414	156.00674
P	3.695	153.37	3.69723	161.03921
S	3.595	137.78	3.59032	173.11715
Ga	3.905	208.69	3.91105	201.29901
Ge	3.813	190.58	3.80414	201.29901
As	3.769	155.38	3.69723	206.33149
Se	3.746	146.33	3.59032	216.39644
In	3.976	301.21	4.08923	276.78614
Sn	3.913	285.12	3.98232	276.78614
Sb	3.938	225.78	3.87541	276.78614
Te	3.982	200.14	3.7685	286.85109
Na	2.658	15.09	2.80099	251.62377
Ca	3.028	119.68	3.0932	25.16238
Fe	2.594	6.54	4.04468	27.67861
Zn	2.462	62.38	4.04468	27.67861
Ti	2.829	8.55	4.04468	27.67861
Tc	2.671	24.14	4.04468	27.67861
Ru	2.64	28.16	4.04468	27.67861

**Table 2:** Lennard-Jones parameters for TraPPE force field.

Atom type	*σ* (Å)	*ε* (K)	Charge
C_co_2_	2.80	27.0	0.700
O_co_2_	3.05	79.0	−0.350
N_n_2_	3.31	36.0	−0.482
N_com	—	—	0.964

All atoms in the MOF were held fixed at their crystallographic positions. The number of unit cells used was different for each MOF to ensure that the perpendicular lengths of the supercell were greater than twice the cutoff used. The cutoff for Lennard-Jones and charge-charge short-range interactions was 12.8 Å and the Ewald sum technique was applied to compute the long-range electrostatic interactions with a relative precision of 10^−6^. The Lennard-Jones potential was shifted to zero at the cutoff. Fugacities needed to impose equilibrium between the system and the external ideal gas reservoir at each pressure were calculated using the Peng-Robinson equation of state^62^ with the critical parameters for each gas taken from Table 3. All GCMC uptake data report the absolute adsorption value in mol/kg units.

**Table 3:** Critical parameters for CO_2_ and N_2_.

Gas	Critical temperature (K)	Critical Pressure (Pa)	Acentric factor
CO_2_	304.1282	7377300.0	0.22394
N_2_	126.192	3395800.0	0.0372

### Lennard-Jones parameters

The Lennard-Jones parameters for DREIDING and UFF force fields used in the calculations for the framework atoms are shown in Table 1. For simplicity, only the atoms that are present in both UFF and DREIDING are shown. The TraPPE parameters used for the gas molecules are present in Table 2. The critical parameters used in the Peng-Robinson equation to calculate the fugacity are present in Table 3.

### Automatic equilibration detection and truncation

To eliminate the use of long initialization cycles, the Marginal Standard Error Rule (MSER)^63^ was applied to automatically detect the ideal truncation point using the pyMSER package v1.0.18 (https://github.com/IBM/pymser), so that the averages were taken only over the equilibrated phase of the simulation. The output of this method is the equilibrated average of the observable, alongside an uncertainty metric. Here we used the uncorrelated standard deviation, as explained in the next sub-section.

The MSER defines the start of the equilibrated region $\widehat{d}(n)$ by solving the minimization problem:

3 $$\widehat{d}(n)=\mathop{{\rm{\arg }}\;{\rm{\min }}}\limits_{0\le k\le n-2}\;{g}_{n}(k)\quad \quad {\rm{where}}\quad \quad {g}_{n}(k)=\frac{1}{{(n-k)}^{2}}\mathop{\sum }\limits_{j=k}^{n-1}{({Y}_{j}-{\bar{Y}}_{n,k})}^{2}=\frac{{S}_{n,k}^{2}}{n-k}$$

The enthalpy of adsorption was computed as

4 $$\Delta H=\frac{\langle U\cdot N\rangle -\langle U\rangle \langle N\rangle }{\langle {N}^{2}\rangle -\langle {N\rangle }^{2}}-RT$$

where *N* is the number of adsorbates on the simulation box and *U* is the potential energy^64^. All the adsorption enthalpy values are reported in kJ/mol and are the values as calculated by the pyMSER package. The Left-most Local Minimum (LLM) version of MSER was used in a batched data with batch size of 5.

### Uncorrelated standard deviation (uSD)

To use an uncertainty metric that reflects, at the same time, the real dispersion of the simulated values and the number of cycles used for this simulation, the *uncorrelated standard deviation* (uSD) was used as an uncertainty metric. To calculate this quantity, first the number of uncorrelated states in the simulation is estimated by calculating the *autocorrelation time*. The autocorrelation time is estimated by calculating the autocorrelation function of the equilibrated data. An exponential decay function is fitted over the values of the autocorrelation function and the autocorrelation time is calculated as the half-life of this exponential decay.

The equilibrated data is then divided into chunks so that each chunk has a number of data points equivalent to the *autocorrelation time*. Then, the average value of each chunk is calculated and the standard deviation over this list of uncorrelated average values is calculated as the uSD. The uncorrelated standard deviation was obtained, as described above, using the pyMSER package.

### Automatic simulation workflow

A set of scripts composed of three stages were created to automate the isotherm generation process. First, a pre-processing step is performed where partial charges are calculated for all structures. Next, a set of calculation scheduler scripts are executed, where steps such as copying the force field and CIF files, creating a supercell with P1 symmetry, writing the RASPA input file, running the RASPA simulation, parsing the RASPA output, and performing the MSER analysis of the results for averaging over the equilibrated phase of the simulation, are run in sequence.

**Fig. 1:**
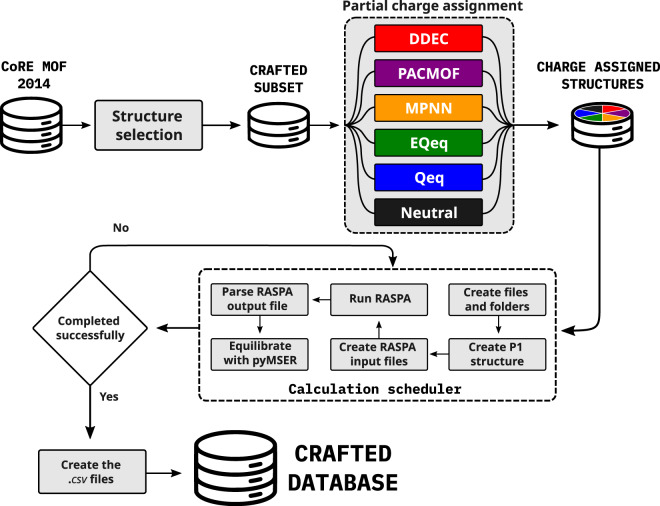
Schematic representation of the main steps in the automated simulation workflow.

Finally, a post-processing script is executed to analyze the results and resubmit incomplete calculations that exceeded the time allocated for the job submission queue. Whenever necessary, the simulations were resumed from a restart binary file generated by RASPA every 1000 cycles. A simplified scheme containing the main steps of this workflow is present on Fig. 1.

### Revised autocorrelations (RACs) descriptors

To understand the diversity of our subset of CRAFTED structures, and understand how representative they are with respect to the MOF material class, revised autocorrelations (RACs) descriptors were calculated using the molSimplify software v1.7.1^65^. RACs are built by generating a crystal graph derived from the adjacency matrix computed for the primitive cell of the crystal structure and calculating the discrete correlations between atomic properties (Pauling electronegativity, nuclear charge, etc.) over the atoms. The correlations are composed of the products (Equation 5) and the differences (Equation 6) of an atomic property *P* for atom *i*, which is selected from the *start* atom list and is correlated to atom *j* selected from the *scope* atom list when they are separated by *d* number of bonds.

5 $${}_{scope}^{start}{P}_{d}^{prod}=\mathop{\sum }\limits_{i}^{start}\mathop{\sum }\limits_{j}^{\,scope}{P}_{i}{P}_{j}\delta ({d}_{i,j},d)$$

6 $${}_{scope}^{start}{P}_{d}^{diff}=\mathop{\sum }\limits_{i}^{start}\mathop{\sum }\limits_{j}^{\,scope}({P}_{i}-{P}_{j})\delta ({d}_{i,j},d)$$

Six atomic properties were used to compute RACs: atom identity (I), connectivity (T), Pauling electronegativity (*χ*), covalent radii (S), nuclear charge (Z) and polarisability (*a*). These properties are used to generate metal-centred, linker and functional-group descriptors. To generate a fixed length descriptor, the averages of these descriptors were used, thus generating 156 features (40 for metal chemistry, 68 for linker chemistry and 48 for functional group chemistry) for each MOF structure.

### Dimensionality reduction and cluster analysis

To reduce the dimensionality of the feature vectors that describe CRAFTED structures, the t-Stochastic Neighbour Embedding (t-SNE)^66^ method was employed as implemented in the scikit-learn^67^ package v1.2.1. Different fitting parameters where used for each set of descriptors for metal chemistry (*perplexity* = 45, *early_exaggeration* = 1, *learningrate* = 50), linker chemistry (*perplexity* = 100, *early_exaggeration* = 1, *learningrate* = 50), functional groups chemistry (*perplexity* = 50, *early_exaggeration* = 1, *learningrate* = 200) and geometric properties (*perplexity* = 50, *early_exaggeration* = 1, *learningrate* = 200). The value 5792 was used as a random seed in all analyses.

For the t-SNE projections of the geometric properties, 14 features were used: largest included sphere (D_is_), the largest free sphere (D_fs_), largest included sphere along a free path (D_isfs_), volumetric accessible area (ASA_m2/cm3_), gravimetric accessible area (ASA_m2/g_), volumetric non-accessible area (NASA_m2/cm3_), gravimetric non-accessible area (NASA_m2/g_), unit cell volume, crystal density, accessible volume fraction (AVF), non-accessible volume fraction (NAVF), accessible volume (AV_cm3/g_), non-accessible volume (NAV_cm3/g_), and the number of pockets (n_pockets_). All these properties were calculated using Zeo++ v0.3^68^. The chemical descriptors used for the t-SNE projections of the MOF structures were described in the previous section. All structures that could not have their descriptors calculated were removed from the list.

For the unsupervised cluster analysis, we used the Density-Based Spatial Clustering of Applications with Noise (DBSCAN)^69^ method as implemented in scikit-learn. The DBSCAN analysis was performed using the standardized and scaled set of descriptors with *eps* = 0.09 and minimum number of samples per cluster of 25.

## Data Records

The CRAFTED dataset^59^ is available in a dedicated Zenodo repository at 10.5281/zenodo.7689919. All the simulations were executed in a traditional HPC cluster with a heterogeneous assortment of Intel^®^ Xeon^®^ 2nd generation processors. Each simulated isotherm took on average ~11 CPU-hours to compute, which translates to ~62 CPU-years to generate the full dataset.

CRAFTED provides 49,680 isotherm files and 49,680 adsorption enthalpy files resulting from the GCMC simulation of two gases (CO_2_ and N_2_) on 690 MOF structures at three temperatures (273, 298, and 323 K) using two force fields (UFF and DREIDING) and six partial charge methods (no charges, Qeq, EQeq, PACMOF, MPNN, and DDEC). Alongside the isotherm data, the charge-assigned CIF files, force field and molecule definition files are provided, to ensure reproducibility and facilitate a future database expansion.

Each isotherm file corresponds to a comma-separated value (CSV) file containing three labeled columns corresponding to pressure (in Pa), uptake volume and its uncertainty (in mol/kg). The file names follow the pattern Q_MOF_FF_GAS_T.csv, therefore the isotherm file named DDEC_ABUWOJ_UFF_CO_2__273.csv contains the data corresponding to the CO_2_ adsorption isotherm at 273 K on the ABUWOJ MOF with DDEC partial charges using the UFF force field. The adsorption enthalpy file names follows the same pattern.

The adsorption enthalpy files correspond to a CSV file containing three labeled columns corresponding to pressure (in Pa), adsorption enthalpy and its uncertainty (in kJ/mol), following the same naming pattern as the isotherm files.

The RASPA input file names (Q_MOF_FF_GAS_T.input) follows the same pattern as the isotherm files, the CIF files are separated into folders according to their partial charge (Qeq, EQeq, DDEC, PACMOF, MPNN and NEUTRAL) type and the force field files are separated into folder according to their type (UFF and DREIDING).

## Technical Validation

### Chemical and geometrical diversity of CRAFTED structures

The selection of a subset of structures that can be modeled simultaneously by both UFF and DREIDING force fields may impose a limitation on the structural and chemical representativeness of the CRAFTED database and, consequently, on the results obtained with this data. To ensure that CRAFTED structures form a group that represents the great diversity of experimentally realised MOFs, both the geometrical and chemical diversities must be represented.

To evaluate the geometrical diversity, the pore size (such as the largest included sphere, largest free sphere and largest included sphere along a free path), void fraction, density, unit cell volume, specific area (both gravimetric and volumetric), pore volume (both gravimetric and volumetric) and the number of pockets (non accessible pores) were used as the descriptors for t-SNE projection. Both the accessible and non-accessible specific area and pore volume was used.

For the chemical diversity, the revised autocorrelations (RACs) descriptors^70^ were used. This approach has been successfully applied to study transition metal chemistry^71^ and the chemical diversity of MOF datasets^72^. The chemical characteristics of the MOFs were divided into three categories: metal node chemistry, organic linker chemistry and functional groups chemistry.

**Fig. 2:**
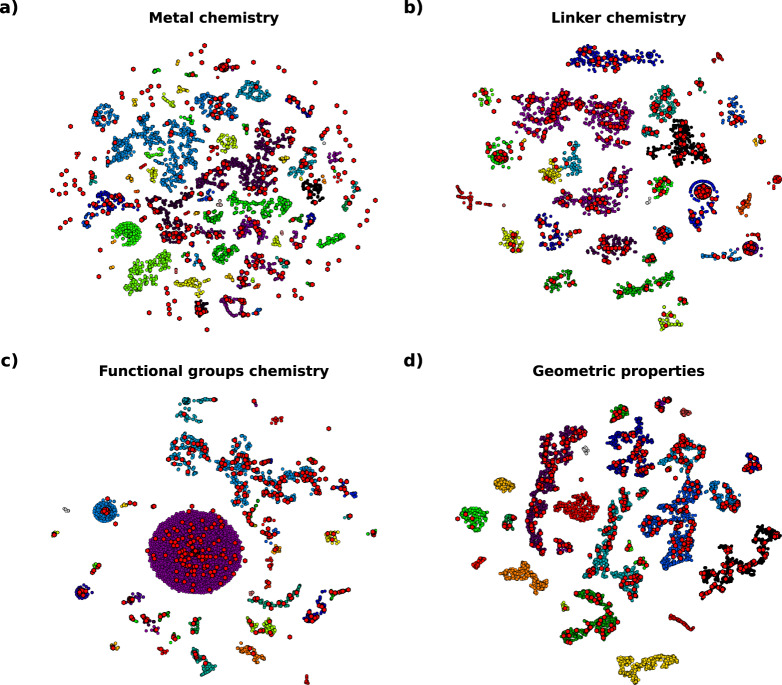
t-SNE representation of the CRAFTED structures (red points) and the CoRE MOF 2019^20,21^ (coloured points) database on different domains of MOF chemistry based on RACs descriptors and geometric properties. (**a**) metal chemistry, (**b**) linker chemistry, (**c**) functional groups chemistry, (**d**) geometric properties. The color scheme correspond to the cluster assigned by DBSCAN.

Figure 2 shows the t-SNE projection onto 2D maps of the four selected groups of descriptors for the structures in the CoRE MOF 2019 database^20,21^ (colored circles) and the selected structures for CRAFTED database (red hexagons). Although CRAFTED structures were taken from the first version of CoRE MOF, from 2014, here the comparison is made with the second version of this database, from 2019, as it has a greater number and diversity of structures.

To numerically evaluate the overlap of the databases in t-SNE projected space, the DBSCAN method was used. This method is an unsupervised machine learning technique used to identify clusters of varying shape and size, grouping points that are close to each other. Since the t-SNE method reduces the feature space by modelling structures with similar features as nearby points and distinct features as distant points, the groups found by the DBSCAN method will share similarities within the original feature space.

The limitation imposed by the DREIDING force field reduces the diversity of metal chemistry observed in the CRAFTED database compared to the structures presented in CoRE MOF 2019, as shown in Fig. 2a. However, 55 of the 95 clusters found (58%) have some structure present in CRAFTED, indicating that even with a limited metal cluster composition, the CRAFTED structures show a good representation of the chemical diversity present in CoRE MOF 2019.

The chemical diversity of both organic linker and functional groups is much better represented within the CRAFTED structures, as can be seen in Fig. 2b,c. In both cases, one can see that the points from both CRAFTED and CoRE MOF 2019 structures are equally dispersed across 2D space. Additionally, 32 of the 35 clusters identified for linker chemistry (91%) and 40 of the 43 clusters identified for functional group chemistry (93%) contain structures present in CRAFTED.

**Fig. 3:**
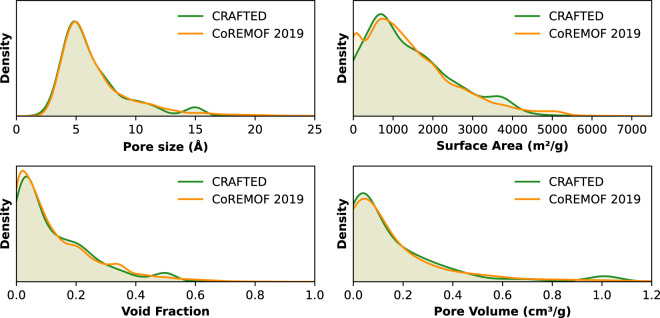
Comparison between the main geometric properties of the structures present in CRAFTED and CoRE MOF 2019^20,21^.

The geometric properties are also well represented by the CRAFTED database, as shown in Fig. 2d. From the 27 clusters identified, 20 (74%) present structures from CRAFTED. Additionally, Fig. 3 shows the distribution density of the values for the main geometric properties presented by the structures on CRAFTED and CoRE MOF 2019. One sees that both databases contain similar distributions, showing that even with a smaller number of structures, the CRAFTED database is exemplary of synthesized MOF structural properties.

### General impact of force field and partial charge selection

**Fig. 4:**
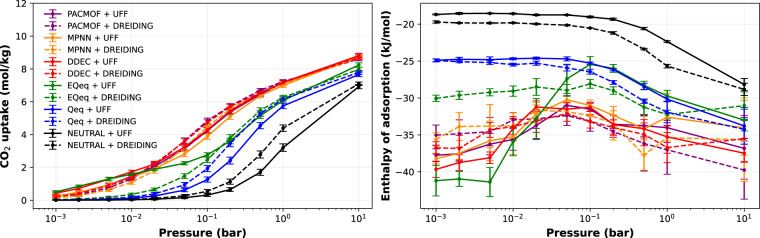
Example of the impact of the choice of force fields and partial charges on the simulated (**a**) adsorption isotherms and (**b**) enthalpy of adsorption of CO_2_ on MULQOA MOF at 273 K.

To illustrate the impact of molecular-level simulation parameters (force field and partial charge) on the outcome of the GCMC simulations, we show in Fig. 4 the absolute uptake and enthalpy of adsorption of CO_2_ on the MULQOA MOF at 237 K. At 0.1 bar, a typical pressure used in adsorption-based pressure swing adsorption (PSA) processes for CO_2_ capture^73^, the uptake values range from 0.5 to 5.0 mol/kg and every combination of partial charge and force field yields a different value, while at 10 bar almost all conditions resulted in similar uptake. This dispersion of results is also reflected on the enthalpy of adsorption that ranges from −17 to −45 kJ/mol and are fairly different for every combination of parameters.

**Fig. 5:**
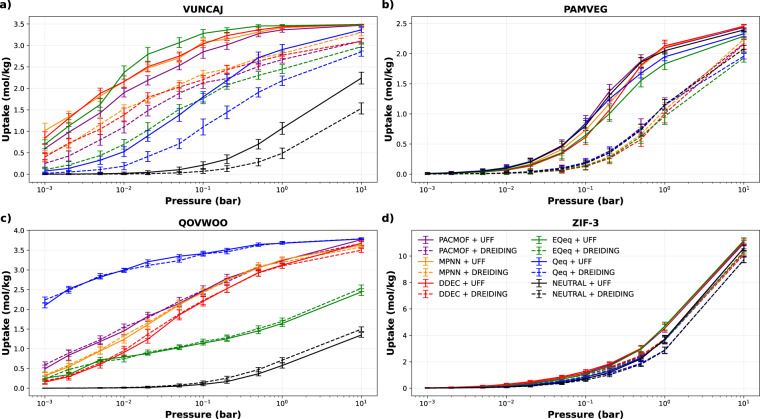
Examples of four representative behaviours found in the dataset: (**a**) high sensitivity to force field and partial charge, (**b**) high sensitivity to force field and low sensitivity to partial charge, (**c**) low sensitivity to force field and high sensitivity to partial charge, and (**d**) low sensitivity to force field and partial charge.

Among the CRAFTED materials, one finds a diversity of responses to the choices of force field and partial charge method. Four representative cases are highlighted in Fig. 5. For some materials, the uptake is highly dependent on both the force field and partial charge, others are only sensitive to one of these parameters, and some are not sensitive to any. Therefore it is possible to anticipate that most studies that depend directly on the results of these molecular simulations, such as high-throughput computational screening or multiscale processes modelling, may also present different degrees of dependence on the choice of parameters.

## Usage Notes

The CRAFTED database contains 49,680 CSV files with the isotherm adsorption data (pressure, uptake, and uncertainty), definition files for UFF and DREIDING force fields, and a total of 4,140 CIF files combining 690 structures with six partial charges (NEUTRAL, DDEC, PACMOF, MPNN, EQeq, and Qeq). The database also contains 49,680 files containing the adsorption enthalpy, and two CSV files containing the set of RAC and geometrical descriptors calculated with molSimplify and Zeo++, respectively. The 49,680 RASPA input files are provided to facilitate the reproduction of the isotherm simulations.

**Fig. 6:**
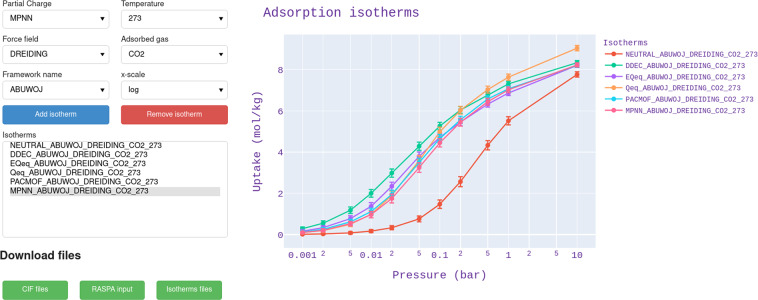
Screenshot of the interactive interface for isotherm visualization.

To facilitate the exploration and visualization of the isotherms and enthalpies of adsorption present on CRAFTED, we also developed an interactive visualization interface based on Jupyter notebooks and panel (v0.14.3). This interface allows the user to select a set of conditions–e.g. partial charge, temperature, force field, adsorbed gas, and material name–for each isotherm, thus facilitating a quick and easy visual comparison of the data. In addition, it is possible to download the CIF files, the inputs for the GCMC simulation with RASPA, and the data of the selected isotherms, thus facilitating the reproduction of the results present in CRAFTED. An example of the interface is shown in Fig. 6. We recommend the user to set up a Python environment using the environment.yml file provided therein.

A Jupyter notebook with the code to perform the t-SNE dimensionality reduction and the DBSCAN unsupervised clusterization analysis with the necessary files containing the RAC and geometric descriptors for the CoRE MOF 2019 is also provided alongside the CRAFTED data, providing an easy reproduction of the results presented in Fig. 2. Please note that, despite having frozen the t-SNE input values, random number generator seed and dependency versions, we observe minor discrepancies in the results when running on different CPU architectures, but which do not alter their interpretation. The results presented herein refer to Intel^®^ x86_64 running Fedora Workstation 37.

To extend the application of the data present on CRAFTED, we prepared a Jupyter notebook with codes that apply the Ideal Adsorbed Solution Theory (IAST) to estimate the CO_2_/N_2_ mixture adsorption uptake and selectivity. We leveraged the pyGAPS package^74^ (v4.4.2) to model the pure component isotherms and predict the multi-component mixture isotherms.

Finally, we would like to highlight some points to show why this database benefits the scientific community. Machine learning (ML) and data-driven methods have become useful tools to aid in the discovery of new materials for CO_2_ capture. For example, surrogate models can be constructed from GCMC-simulated adsorption data to map the structure-property relationship of nanoporous materials, which may then be used to accelerate the HTCS of previously unexplored databases of adsorbents^75,76^. Deep generative models can be trained with simulated adsorption data to develop property-orientated generative algorithms to discover new materials on the latent chemical space optimized for gas capture applications by an inverse design approach^77–79^.

As ML model prediction accuracy and data quality are intrinsically related, there is an apparent requirement to assess the impact of uncertainty from molecular-level simulations on the confidence of machine learning model predictions^80^. The efficacy of the surrogate models, for example, depends on the quality of information provided by the material feature vector, and so concerted efforts have been made to develop useful representations of MOFs^81^. However, little is known about the impact of force field selection on the interpretability of surrogate model predictions. Particularly in the case of MOFs with coordinatively unsaturated metals, different generic force fields and charge assignment schemes can deliver dramatically different results.

Therefore, the importance of material features–learned either explicitly by the surrogate model as in decision trees, or extracted through feature permutations/SHAP values^82^–may be subject to considerable discrepancies. Feature importance analyses are useful to guide the design of new functional MOFs, and so it is desirable to understand the differences (if any) that arise from different levels of molecular theory.

## Data Availability

The Jupyter notebooks providing the panel visualisation of the isotherm curves, enthalpy of adsorption data, IAST-based multicomponent mixture isotherm, and the t-SNE + DBSCAN analysis of the chemical and geometric properties of MOFs are distributed alongside the database in the Zenodo^59^ repository. A fully automated workflow that is capable of recreating the dataset was made available as an open-source project (v1.0.0) on GitHub (https://github.com/st4sd/nanopore-adsorption-experiment).
